# Insurance optimizes complex interactive and cooperative behaviors in public goods games

**DOI:** 10.1371/journal.pone.0197574

**Published:** 2018-05-18

**Authors:** Jinming Du

**Affiliations:** 1 Liaoning Engineering Laboratory of Operations Analytics and Optimization for Smart Industry, Northeastern University, Shenyang 110819, China; 2 Institute of Industrial and Systems Engineering, Northeastern University, Shenyang 110819, China; Rice University, UNITED STATES

## Abstract

Global cooperation is urgently needed to prevent risks of world-wide extreme events and disasters for sustainable development. In human societies, however, there exists bias toward interacting with partners with similar characteristics, but not contributing globally. We study how complex interactive behaviors evolve under risks through proposing a threshold public goods game model. In the model, individuals either play games with participants who own the same phenotype, or contribute to the collective target of global public goods. We further introduce an insurance compensation mechanism into the model to probe the evolution of global cooperation. It is found that the introduction of the insurance remarkably promotes the emergence of global cooperative behavior and inhibits the tendency to play games only with individuals of the same phenotype. Besides, contrary to models without insurance, global cooperation is strengthened with the increase of imitation intensities. In addition, high risk and high threshold are in favor of global cooperation.

## Introduction

Sustainable development calls for global cooperation, which has been attracting more and more focus against the background of globalization [1]. In particular, world-wide cooperation is necessary for solving global social dilemmas, such as over exploitation of natural resources and human-induced climate change [2–7]. The core feature of such situations could be modeled using a public goods game (PGG) [8, 9]. People all around the world have no choice but to be involved in such games, which have been identified as the greatest PGGs of seven billion participants [10]. More than that, these are all the games that we cannot afford to lose [11]. Taking global warming for example, participants face a dilemma [12–14]. Managing the risks of extreme disasters to advance climate change adaptation will surely sacrifice short-term economic development. However, in case that the already-rising global temperature passes a certain threshold, ecological crisis may occur. This will result in substantial human and financial losses. To prevent such risks of danger, we are obligated to reduce the emission of greenhouse gases to 30–60% of the 2010 level by 2050 [15]. This reduction level is so huge that it is impossible for a single participant to manage. Hence, people should sacrifice personal benefits for the common goods and work together to achieve what they are unable to achieve alone. It is necessary to study how to cooperate more or better with others in such collective-risk social dilemmas [13, 16–21]. The problem, however, is not actually as natural as it looks. Cooperation is costly, and performing such an altruistic behavior can bring a huge burden and loss to the individual. Since free riders can also gain the same benefits, what is the motivation for cooperators to contribute to the public goods under the natural law of the fittest survival? Given that cooperation is the basis for our sustainable development and better future, understanding cooperative behaviors in complex interactive systems has been one of the grand scientific challenges of the global society [22–24].

In daily life, it is common that individuals preferentially donate to partners sufficiently similar to themselves in some arbitrary characteristics [25]. Such a characteristic, or generally regarded as a tag, can be a marking, display, phenotype, group, set or other observable traits [26–30]. This kind of similarity-mediated interaction provides criteria based on which individuals select their partners. Hence, we propose a PGG model in which individuals own diverse phenotypes (tags). Individuals with the same phenotype play PGG where they contribute to local affairs, such as regional economics. Otherwise, the players can choose another behavior with great foresight. Out of concern for the global sustainable development, they devote themselves to global public goods for climate change control. In contrast to classical PGG, the climate game involves a threshold. This threshold means that payoffs of individuals are conditional in the sense that they do not vanish if and only if the amount of global public goods exceeds this critical value.

It is well known that the progress in reducing global greenhouse gases emission has been remarkably slow owing to free-riding incentives. Previous research reveals that pledges can increase contributions [5]. In the real world, however, its promotion to cooperation is not as obvious as it is in the literature. Actually, the experience over the past decades shows that the pledge and protocols [31] are minimally effective, which only give people little confidence in the prospect of future. There needs a mechanism of incitement to promote inter-governmental and inter-organizational cooperation, but not pure voluntariness. Global cooperators should be assured that their donation indeed takes effect on the possession protection from risks, whatever actions may be taken by others. Insurance is a means of protection from financial loss. It is a form of risk management primarily used to hedge against the risk of a contingent, uncertain loss. In reality, buying insurance has become a business practice of smart corporations and individuals in order to reduce their own operational risks. An individual who buys insurance is known as an insured. An entity which provides insurance is known as an insurance company. The insurance transaction involves the insured paying a small premium to the insurance company in exchange for the insurance company’s promise to compensate the insured in the event of a covered loss. Since more and more risk events have increased and a variety of disasters become increasingly serious, insurance has become a significant tool for effectively managing risks.

Motivated by this, we introduce a kind of cooperative risk insurance mechanism into the threshold PGG model. The operation of the cooperative risk insurance is as follows: when participating in the threshold PGG, global cooperators are automatically insured in the process of contributing to global public goods. In accordance with the contract agreed conditions, such as the collective target fails and disaster strikes, the insured can obtain a certain percentage of compensation from the insurance company to avoid suffering total loss of property while the property of other strategy holders will be totally lost; on the contrary, if no disaster occurs, all the players obtain their payoffs in the PGGs. Based on this model, we investigate how the insurance compensation mechanism affects the evolution of global cooperation.

## Model

We study the evolutionary process of a threshold public goods game, and introduce an insurance compensation mechanism. In this game, we assume that there exist *M* phenotypes in the whole population with size *N*. Then, each kind of phenotype is held by *m* = *N*/*M* players. The details of the game are as follows. Each individual is given a single unit of money. Individuals either play games with participants who own the same phenotype, or contribute to the collective target of global public goods. The strategy of each individual is denoted as *S*_*k*_ ∈ {*S*, *L*, *G*}. Here *S*, *L* and *G* represent selfishness, local cooperation and global cooperation, respectively. The money of selfish players is saved for herself when playing PGGs with other players with the common phenotype. The local cooperators in such local PGGs donate money into a local account. Then the total amount is multiplied by a local gain-factor *r*_1_ (1 < *r*_1_ < *m*), and equally distributed to the individuals who participate in the focal game. For global cooperators, their donations for global public goods are summed, and multiplied by a global gain-factor *r*_2_ (*r*_1_ < *r*_2_ < *N*). Then the global public goods are shared by all the individuals in the whole population irrespective of whether they are global cooperators or not. The payoff of each individual is threatened by the global risk which is depicted by a threshold *s*. The global public goods are utilized to prevent disasters. If the amount of global public goods is greater than *s*, the collective target achieves and disasters are not going to happen. In this case, all the players will get their payoffs in the public goods games. If the amount of collected global public goods is less than *s*, the disaster would happen with a probability *q*. Once a disaster happens, the property of all the players will be lost. However, global contributors can get some compensation from the insurance. Here, we denote *ϕ* (0 < *ϕ* < 1) as the rate of insurance compensation. Thus, global contributors can take back such percentage of their donations. We use pairwise comparison as the strategy updating rule. We randomly choose two individuals, namely *A* and *B*, respectively. With probability $1/[1+e^{-\omega (\pi_{B}-\pi_{A})}]$, *A* learns *B*’s strategy [32–36], where *π*_*k*_ is the payoff of individual *k* and *ω* is the imitation intensity. We consider two imitation intensities: *ω*_1_ as the imitation intensity between players with the same phenotype; *ω*_2_ as the imitation intensity between players with different phenotypes. Besides, with a probability *μ* (*μ* → 0), individuals could change its strategy to another one.

## Results and discussion

**Fig 1:**
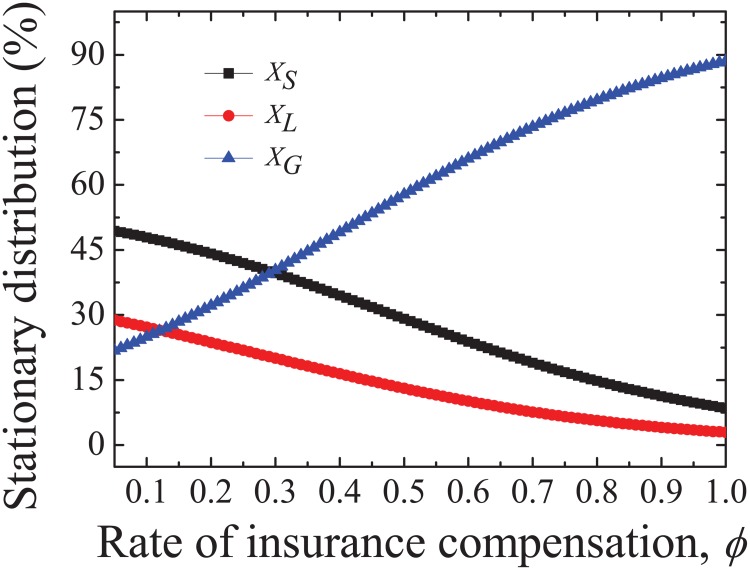
Stationary distribution changes with the increase of insurance compensation rate *ϕ*. As is shown in the figure, *X*_*G*_ is promoted with the increasing *ϕ*, while *X*_*L*_ and *X*_*S*_ are inhibited. It means that players are more apt to cooperate in the case of more guarantee. Parameter values are *N* = 100, *M* = 20, *q* = 0.8, *r*_1_ = 2, *r*_2_ = 3, *s* = 200 and *ω*_1_ = *ω*_2_ = 0.05.

In this paper, we study how global cooperative behavior is affected by collective risk and insurance compensation mechanism. It is found that global cooperation booms with the increase of insurance compensation, while participants taking part in games with the same phenotype holders are inhibited. As is illustrated in Fig 1, the stationary distribution of *G* (*X*_*G*_) increases while *X*_*L*_ and *X*_*S*_ decrease. As distinct from classical threshold PGG, *G* strategy here has an implicit income after the insurance compensation is introduced. If we consider a PGG among players with a same phenotype in which there are *i* *G* players, *j* *L* players and *m* − *i* − *j* *S* players. We further assume that all the other players in the whole population are *S* players. Denote *π*_*G*_(*i*), *π*_*L*_(*j*) and *π*_*S*_(*m* − *i* − *j*) as the payoff of each *G*, *L* and *S* player, respectively. In the PGG, we have *π*_*G*_(*i*) = *i* × *r*_2_/*N* + *j* × *r*_1_/*m*, *π*_*L*_(*j*) = *i* × *r*_2_/*N* + *j* × *r*_1_/*m* and *π*_*S*_(*m*−*i*−*j*) = *i* × *r*_2_/*N* + *j* × *r*_1_/*m* + 1. Considering the risks, we introduce the threshold function *θ* = *q* when *ir*_2_ < *s* and *θ* = 0 when *ir*_2_ ≥ *s*. Once disaster strikes, all the individuals lose their payoffs except global cooperators. Owing to the insurance compensation mechanism, the insurance company will pay for the loss of global cooperators. Therefore, the global cooperator’s payoff should be superadded an additional part. Then we have: *π*_*G*_(*i*) = *ϕθ* + (*i* × *r*_2_/*N* + *j* × *r*_1_/*m*)(1−*θ*), *π*_*L*_(*j*) = (*i* × *r*_2_/*N* + *j* × *r*_1_/*m*)(1−*θ*), and *π*_*S*_(*m*−*i*−*j*) = (*i* × *r*_2_/*N* + *j* × *r*_1_/*m* + 1)(1−*θ*). Hence, the payoff of *G* would be larger than those of *L* and *S* when disaster happens. Such hidden income changes the payoff expectation of global cooperators and also the comparison among strategies. The effect of insurance compensation becomes remarkably obvious, especially under the high risk of danger.

We then address how global cooperative behavior is affected by risks. By adding a threshold, the game is turned from a multi-person prisoner’s dilemma to a kind of coordination game. In particular, in contrast to a linear public goods game, the threshold public goods game contains at least one cooperative equilibrium. In such condition, if all the other players contribute, the focal player should also contribute from a rational and selfish point of view. In our model, it is found that global cooperation is promoted provided that individuals realize that they are facing a sufficiently severe potential crisis. *X*_*G*_ increases with the rise of the danger probability *q* and also the threshold *s*. High risk means that all the players probably lose their wealth. Then global cooperators can gain a foothold owing to the compensation in such case. This paves the way for them to dominate the population. Global cooperation is necessary for public safety, and becomes more and more important with the increasing threshold. High threshold means a bigger target which has to be reached to avoid the risk. It hints that individuals are inclined to cooperate with others to resist the disaster, since any single one can’t afford the huge expense.

**Fig 2:**
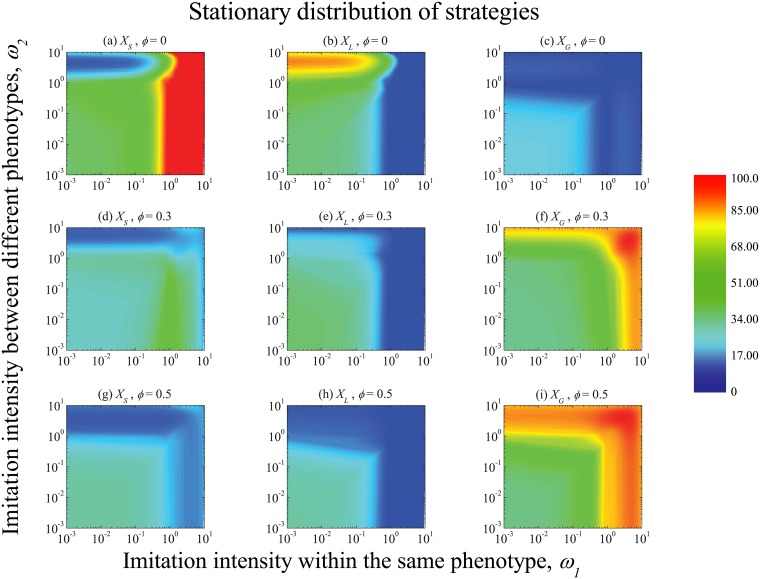
The influence of imitation intensities on the stationary distribution. Without insurance compensation, as shown in panel (*c*), the global cooperation decreases with the rise of *ω*_1_ and *ω*_2_. When *ω*_1_ ascends, as shown in panel (*a*), the proportion of selfish individuals increases while that of local cooperators decreases. When *ω*_2_ ascends, as shown in panel (*b*), the fraction of local cooperators increases. However, *X*_*G*_ increases with the rise of imitation intensities after introducing insurance compensation, which are shown in panel (*f*) and (*i*). It indicates that global cooperative behavior can emerge in the threshold PGG with an insurance compensation mechanism when the imitation intensities are strong. It depicts the situation that under high risk and high threshold conditions, players face a high probable crisis in which global cooperators may be promoted. Parameter values are *N* = 100, *M* = 20, *r*_1_ = 2, *r*_2_ = 3, *q* = 0.8 and *s* = 200.

Subsequently, the influences of different imitation intensities are shown in Fig 2. It is depicted that the global cooperation decreases with the rise of *ω*_1_ and *ω*_2_ when there is no insurance compensation. It indicates that global cooperative behavior can emerge only if the imitation intensities are sufficiently weak. When the imitation intensity between the same phenotype holders *ω*_1_ ascends, the proportion of selfish individuals increases while that of local cooperators decreases. However, when the imitation intensity between players with different phenotypes *ω*_2_ ascends, the fraction of local cooperators increases. Thus, the strong between-phenotype imitation intensity is in favor of local cooperators. Actually, when the two imitation intensities are both weak, the dynamics of each strategy is mainly driven by random drift. Thus each of the three is around 1/3 of the whole population. When the imitation intensity increases (either of the two), global cooperation is the worst strategy. By introducing the insurance compensation, however, such situation is changed. Global cooperative behavior emerges under stronger imitation intensities. This differs from the results in classical PGG model and threshold PGG model without insurance compensation. The compensation mechanism leads to the change of payoff expectation of strategies in the game. *G* has been no longer always inferior, since it has a part of implied potential payoff. Under high risk and high threshold conditions, players face a high probable crisis in which global cooperators may possess larger payoffs. Thus, global cooperation is more likely to be selected under stronger imitation intensities.

**Fig 3:**
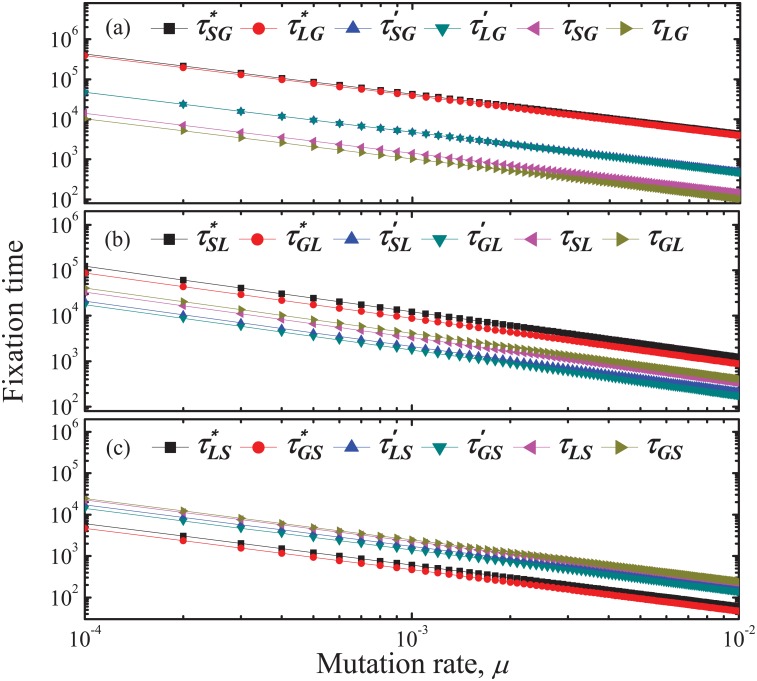
The fixation time as a function of the mutation rate *μ*. With the rise of *μ*, fixation time needed becomes shorter. In each panel, fixation time of strategies in threshold PGG models with an insurance compensation and without one, and the standard PGG model without a threshold are respectively shown and compared. (*a*) A mutant *G* invades *S* and *L* population. *τ*_*SG*_ and *τ*_*LG*_ are the average time starting in pure state of *S* and *L* to reach *G*, respectively, in the threshold PGG model with an insurance compensation. Likely, $\tau_{SG}^{\prime }$ and $\tau_{LG}^{\prime }$ are the fixation time for the threshold PGG model without an insurance compensation. Besides, $\tau_{SG}^{*}$ and $\tau_{LG}^{*}$ are the fixation time for the standard PGG model without a threshold. It is shown that *τ*_*SG*_ (and *τ*_*LG*_) are shorter than $\tau_{SG}^{\prime }$ (and $\tau_{LG}^{\prime }$) and $\tau_{SG}^{*}$ (and $\tau_{LG}^{*}$). (*b*) A mutant *L* invades *S* and *G* population. (*c*) A mutant *S* invades *L* and *G* population. Comparing the three panels, it is shown that the fixation time of *G* strategy is longer than that of *L* and *S*, and the fixation time of *S* is the shortest in the same model. The introduction of threshold and insurance compensation into PGG evidently shortens the fixation time of global cooperation strategy. Parameter values are: *N* = 100, *M* = 20, *r*_1_ = 2, *r*_2_ = 3, *s* = 200, *q* = 0.8, *ϕ* = 0.5 and *ω*_1_ = *ω*_2_ = 0.05.

Finally we study the fixation time of strategies. It is found that the fixation time changes a lot, especially that of *G* strategy, after introducing a threshold and further an insurance compensation into the PGG model. The average time that a mutant of each strategy invades population full of the other two respectively are shown in Fig 3. The time for *G* invading *S* is the longest, while the time for *S* invading *G* is the shortest. Owing to inserting a threshold, the time for *G* strategy invading the others is obviously shortened. By introducing an insurance compensation mechanism, the time is further contracted. While, the change of the fixation time of *S* is on the contrary. It is noted that the fixation time of *L* is sharply shortened in the threshold PGG compared with classical PGG, while it rises after introducing an insurance compensation. Hence, the introduction of threshold and insurance compensation into PGG evidently affects the fixation time of strategies. To a certain degree, both of the two mechanisms promote the emergence of global cooperative behaviors.

## Conclusions

In conclusion, our research sheds light on that crisis awareness can promote global cooperation. When individuals are conscious of an even greater calamity, they are apt to form an alliance through cooperation to prevent the risk. The more disruptive the danger is, the more likely they succeed in cooperation. Therefore, raising all the people’s risk awareness of various kinds of catastrophe could be beneficial to promoting global investment for sustainable development. Moreover, our results imply that an insurance compensation mechanism may be effective for solving global social dilemmas. Such mechanism appears like a reward or incitation to players in the game. It makes the global cooperator’s behavior no longer be enslaved to other players’ choice. It also offers global cooperators more psychological guarantee for their possession, because their donation for preventing risks will prospectively reduce their potential loss to a certain extent. It thus might heighten their confidence in global cooperative behaviors.

The fund of insurance compensation may be not only made up of global cooperators’ insurance premium, but also other sources, such as government’s financial assistance, organizations or companies’ donation, or income of the insurance company through its other insurance services. The insurance company or other insurance fund management institutions do not take part in the public goods game, but collects money (insurance premium) from players who buy insurance, and provides compensation to those insured when disaster happens. For example, in response to flood risk, UK proposes a flood insurance plan. As a non profitable flood insurance fund, such plan provides insurance for the households with the highest flood risk by utilizing 180 million pounds of tax. Once it rains more than a certain days during a time span and influence people’s everyday life, the insured people would obtain compensations. Besides, a variety of risk insurance fund have been taken into practice around the world. For instance, in a proposal for an “international insurance pool” within the United Nations Framework Convention on Climate Change (UNFCCC) context, the Alliance of Small Island States (AOSIS) put forth the idea of a global compensation fund fully financed by industrialized countries for the purpose of compensating low-lying States for sea-level rise damages. The fund is characterized by voluntary, media-driven and uncoordinated donations. The AOSIS insurance proposal addressed the gradual onset of sea-level rise, thus subsequent proposals have turned to sudden-onset weather events such as floods, tropical cyclones and sea surges (worsened by sea-level rise). Then a Climate Impact Relief Fund (CIRF) is advocated, which is regularly funded up-front and centrally administered by the UNFCCC in order to increase efficiency and fairness. No “new money” would be needed, since Organization for Economic Co-operation and Development (OECD) countries would donate to the fund proportionally to their current average post-disaster assistance spending. In addition, the risk management of unexpected disasters is rising in the insurance industry. More and more insurance companies are starting to offer risk insurance for people’s personal life, such as tourism climate insurance. All of the above could provide risk insurance funds for cooperative risks of global cooperators. In any case, promoting a large cooperative effect with a relatively small amount of insurance fund, may always be a wise choice.

## References

[1] Robertson R. Globalization: Social Theory and Global Culture. Los Angeles, CA: SAGE; 1992.

[2] Ostrom E, Burger J, Field CB, Norgaard RB, Policansky D. Revisiting the commons: Local lessons, global challenges. Science. 1999;284(5412):278–282. doi: 10.1126/science.284.5412.278 10195886 10.1126/science.284.5412.278

[3] Dietz T, Ostrom E, Stern PC. The struggle to govern the commons. Science. 2003;302(5652):1907–1912. doi: 10.1126/science.1091015 14671286 10.1126/science.1091015

[4] Dreber A, Nowak MA. Gambling for global goods. Proc Natl Acad Sci USA. 2008;105(7):2261–2262. doi: 10.1073/pnas.0800033105 18287079 10.1073/pnas.0800033105 PMC2268122

[5] Tavoni A, Dannenberg A, Kallis G, Löschel A. Inequality, communication, and the avoidance of disastrous climate change in a public goods game. Proc Natl Acad Sci USA. 2011;108(29):11825–11829. doi: 10.1073/pnas.1102493108 21730154 10.1073/pnas.1102493108 PMC3141931

[6] Nordhaus W. Climate clubs: Overcoming free-riding in international climate policy. Am Econ Rev. 2015;105(4):1339–1370. doi: 10.1257/aer.15000001

[7] Du J, Wu B, Wang L. Aspiration dynamics and the sustainability of resources in the public goods dilemma. Phys Lett A. 2016;380(16):1432–1436. doi: 10.1016/j.physleta.2016.02.039

[8] Hardin G. The tragedy of the commons. Science. 1968;162(3859):1243–1248. doi: 10.1126/science.162.3859.1243 5699198

[9] Hardin G. Extensions of the “Tragedy of the commons”. Science. 1998;280(5364):682–683. doi: 10.1126/science.280.5364.682

[10] Pfeiffer T, Nowak MA. Climate change: All in the game. Nature. 2006;441(7093):583–584. doi: 10.1038/441583a 16738648 10.1038/441583a

[11] Milinski M, Semmann D, Krambeck HJ, Marotzke J. Stabilizing the Earth’s climate is not a losing game: Supporting evidence from public goods experiments. Proc Natl Acad Sci USA. 2006;103(11):3994–3998. doi: 10.1073/pnas.0504902103 16537474 10.1073/pnas.0504902103 PMC1449634

[12] Kerr RA. Global warming is changing the world. Science. 2007;316(5822):188–190. doi: 10.1126/science.316.5822.188 17431148 10.1126/science.316.5822.188

[13] Milinski M, Sommerfeld RD, Krambeck HJ, Reed FA, Marotzke J. The collective-risk social dilemma and the prevention of simulated dangerous climate change. Proc Natl Acad Sci USA. 2008;105(7):2291–2294. doi: 10.1073/pnas.0709546105 18287081 10.1073/pnas.0709546105 PMC2268129

[14] Du J, Wu B, Wang L. Climate collective risk dilemma with feedback of real-time temperatures. Europhys Lett. 2014;107(6):60005 doi: 10.1209/0295-5075/107/60005

[15] IPCC. Climate Change 2014: Impacts, Adaptation and Vulnerability. Cambridge, UK: Cambridge University Press; 2014.

[16] Buchan NR, Grimalda G, Wilson R, Brewer M, Fatas E, Foddy M. Globalization and human cooperation. Proc Natl Acad Sci USA. 2009;106(11):4138–4142. doi: 10.1073/pnas.0809522106 19255433 10.1073/pnas.0809522106 PMC2657440

[17] Chen X, Szolnoki A, Perc M. Risk-driven migration and the collective-risk social dilemma. Phys Rev E. 2012;86(3):036101 doi: 10.1103/PhysRevE.86.036101 10.1103/PhysRevE.86.036101 23030974

[18] Du J, Wu B, Wang L. Evolution of global cooperation driven by risks. Phys Rev E. 2012;85(5):056117 doi: 10.1103/PhysRevE.85.056117 10.1103/PhysRevE.85.056117 23004831

[19] Hilbe C, Abou Chakra M, Altrock PM, Traulsen A. The evolution of strategic timing in collective-risk dilemmas. PLoS ONE. 2013;8(6):e66490 doi: 10.1371/journal.pone.0066490 23799109 10.1371/journal.pone.0066490 PMC3682992

[20] Chen X, Zhang Y, Huang TZ, Perc M. Solving the collective-risk social dilemma with risky assets in well-mixed and structured populations. Phys Rev E. 2014;90(5):052823 doi: 10.1103/PhysRevE.90.052823 10.1103/PhysRevE.90.052823 25493849

[21] Du J, Tang L. Evolution of global contribution in multi-Level threshold public goods games with insurance compensation. J Stat Mech. 2018;2018(1):013403 doi: 10.1088/1742-5468/aa9bb6

[22] Perc M, Jordan JJ, Rand DG, Wang Z, Boccaletti S, Szolnoki A. Statistical physics of human cooperation. Phys Rep. 2017;687:1–51. doi: 10.1016/j.physrep.2017.05.004

[23] Rand DG, Nowak MA. Human cooperation. Trends Cogn Sci. 2013;17(8):413–425. doi: 10.1016/j.tics.2013.06.003 23856025 10.1016/j.tics.2013.06.003

[24] Perc M. Phase transitions in models of human cooperation. Phys Lett A. 2016;380(36):2803–2808. doi: 10.1016/j.physleta.2016.06.017

[25] Riolo RL, Cohen MD, Axelrod R. Evolution of cooperation without reciprocity. Nature. 2001;414:441–443. doi: 10.1038/35106555 11719803 10.1038/35106555

[26] Traulsen A, Schuster HG. Minimal model for tag-based cooperation. Phys Rev E. 2003;68(4):046129 doi: 10.1103/PhysRevE.68.046129 10.1103/PhysRevE.68.046129 14683024

[27] Blackwell C, McKee M. Only for my own neighborhood? Preferences and voluntary provision of local and global public goods. J Econ Behav Organ. 2003;52(1):115–131. doi: 10.1016/S0167-2681(02)00178-6

[28] Traulsen A, Nowak MA. Chromodynamics of cooperation in finite populations. PLoS ONE. 2007;2(3):e270 doi: 10.1371/journal.pone.0000270 17342204 10.1371/journal.pone.0000270 PMC1803018

[29] Antal T, Ohtsuki H, Wakeley J, Taylor PD, Nowak MA. Evolution of cooperation by phenotypic similarity. Proc Natl Acad Sci USA. 2009;106(21):8597–8600. doi: 10.1073/pnas.0902528106 19416902 10.1073/pnas.0902528106 PMC2688992

[30] Tarnita CE, Antal T, Ohtsuki H, Nowak MA. Evolutionary dynamics in set structured populations. Proc Natl Acad Sci USA. 2009;106(21):8601–8604. doi: 10.1073/pnas.0903019106 19433793 10.1073/pnas.0903019106 PMC2689033

[31] Tollefson J. Last-minute deal saves climate talks. Nature. 2010;468(7326):875–876. doi: 10.1038/468875a 21164449 10.1038/468875a

[32] Blume LE. The statistical mechanics of strategic interaction. Games Econ Behav. 1993;5(3):387–424. doi: 10.1006/game.1993.1023

[33] Szabó G, Tőke C. Evolutionary prisoner’s dilemma game on a square lattice. Phys Rev E. 1998;58(1):69–73. doi: 10.1103/PhysRevE.58.69

[34] Traulsen A, Pacheco JM, Nowak MA. Pairwise comparison and selection temperature in evolutionary game dynamics. J Theor Biol. 2007;246(3):522–529. doi: 10.1016/j.jtbi.2007.01.002 17292423 10.1016/j.jtbi.2007.01.002 PMC2001316

[35] Wu B, Zhou D, Fu F, Luo Q, Wang L, Traulsen A. Evolution of cooperation on stochastic dynamical networks. PLoS ONE. 2010;5(6):e11187 doi: 10.1371/journal.pone.0011187 20614025 10.1371/journal.pone.0011187 PMC2894855

[36] Traulsen A, Semmann D, Sommerfeld RD, Krambeck HJ, Milinski M. Human strategy updating in evolutionary games. Proc Natl Acad Sci USA. 2010;107(7):2962–2966. doi: 10.1073/pnas.0912515107 20142470 10.1073/pnas.0912515107 PMC2840356

